# Clinical and Angiographic Profile of Acute Coronary Syndrome: Insights From a Tertiary Care Center in an Industrial Area

**DOI:** 10.7759/cureus.83805

**Published:** 2025-05-09

**Authors:** Anindya Banerjee, Ishita Majumdar, Madhurima Ganguly, Mohammed I Hussain, Debasish Das

**Affiliations:** 1 Cardiology, Healthworld Hospital Asansol, Asansol, IND; 2 Neonatology, Institute of Post Graduate Medical Education and Research (IPGMER) and Seth Sukhlal Karnani Memorial Hospital (SSKM Hospital), Kolkata, IND; 3 Cardiology, All India Institute of Medical Sciences, Bhubaneswar, Bhubaneswar, IND

**Keywords:** acute coronary syndrome, cad, environmental cardiology, industrial pollution, microvascular dysfunction, minoca, non-obstructive cad, nstemi, scad, stemi

## Abstract

Background

Acute coronary syndrome (ACS) patterns may be modified by environmental factors in industrial regions. This study examines the clinical and angiographic profile of ACS patients in a polluted industrial area of Eastern India.

Methods

We analyzed 213 consecutive ACS patients undergoing coronary angiography at a tertiary care center (April 2024-March 2025). Using RStudio, we performed descriptive statistics, comparative analyses (t-tests/Mann-Whitney U test for continuous variables; chi-square/Fisher's exact test for categorical variables), and multivariable logistic regression adjusting for age, sex, and hypertension. Effect sizes were calculated as odds ratios (OR) with 95% confidence intervals.

Results

The cohort (median age 58 years; 64.8% male) showed a high prevalence of dyslipidemia (119; 55.9%) and hypertension (103; 48.4%), with non-ST-elevation myocardial infarction (NSTEMI) being the most common presentation (86; 40.4%). Angiography revealed obstructive coronary artery disease (CAD) in 170 (79.8%) (predominantly LAD involvement: 85), non-obstructive CAD in 43 (20.2%), and slow-flow phenomenon in 61 (28.6%). Notably, spontaneous coronary artery dissection (SCAD) cases (11; 5.2%) showed an atypical 1:1 sex ratio. Patients with obstructive CAD were significantly older (median 62 vs 44 years, p<0.001) and demonstrated a significantly higher prevalence of diabetes in non-obstructive CAD (unadjusted OR 7.5, 95% CI 3.6-15.7, p<0.001).

Conclusion

This industrial cohort exhibited distinct ACS patterns, including high rates of microvascular dysfunction and atypical SCAD epidemiology, suggesting potential environmental influences on coronary pathophysiology that warrant further investigation.

## Introduction

Acute coronary syndrome (ACS) is a significant contributor to cardiovascular morbidity and mortality worldwide. The etiopathogenesis of both acute and chronic coronary artery disease (CAD) is multifactorial. In the current era of medicine, there has been a paradigm shift toward prevention and modification of risk factors involved. Recent studies have demonstrated that environmental pollutants play an important role in increasing the burden of CAD [1-4].

Registry data from various ACS registries have been exceedingly valuable in understanding the epidemiological and risk factor spectrum of ACS [5-7]. Moreover, these registries have shown that the clinico-epidemiological spectrum of ACS in the subcontinent differs significantly from the Western population. This underlines the significant geographic, ethnic, and environmental variation of the spectrum of ACS.

Research studies have shown a consistent relationship between CAD and environmental pollution [1-4]. Particularly, the occurrence of coronary microvascular dysfunction and myocardial infarction with non-obstructive coronary arteries (MINOCA) in association with long-term exposure to particulate matter seems interesting [4].

This study aimed to look at the clinical and angiographic spectrum of ACS in a tertiary care center in an industrial area from eastern India, where the residing population is constantly exposed to high levels of environmental pollution.

## Materials and methods

Aim

There is limited data on the clinical and angiographic profiles of ACS in industrial regions with high particulate matter exposure. This study aimed to evaluate the clinical and angiographic spectrum of ACS in a tertiary care hospital located in an industrial zone of eastern India.

Study population

We retrospectively included all patients admitted with a confirmed diagnosis of ACS between April 2024 and March 2025. Only those who underwent coronary angiography (CAG) were included in the final analysis. No patients refused CAG or died prior to the procedure. ACS was defined by typical symptoms with electrocardiographic changes and/or elevated cardiac biomarkers.

Data collection

Clinical and angiographic data were extracted from hospital medical records. A total of 213 patients met the inclusion criteria.

Angiographic interpretation

Coronary angiograms were reviewed by two independent interventional cardiologists, blinded to clinical data. Significant CAD was defined as ≥70% stenosis in a major epicardial artery or ≥50% in the left main artery, based on visual estimation. Discrepancies were resolved by consensus.

Handling of missing data

There was no missing data among the included patients. All key clinical, demographic, and angiographic variables were complete.

Statistical analysis

Continuous variables were expressed as mean ± SD (if normally distributed) or median with interquartile range (IQR). Categorical variables were presented as frequencies and percentages. Group comparisons used the student’s t-test or Mann-Whitney U test for continuous variables, and the chi-square or Fisher’s exact test for categorical variables.

Effect sizes were reported as odds ratios (OR) with 95% confidence intervals (CI) for binary outcomes. Non-parametric effect sizes (Hodges-Lehmann median differences) were calculated for skewed data. Multivariable logistic regression, adjusted for age, sex, and hypertension, was used to examine associations with obstructive CAD. Multicollinearity was excluded (all VIF <2). A two-tailed p-value <0.05 was considered statistically significant. Analyses were conducted using RStudio (RStudio Team (2020). RStudio: Integrated Development for R. RStudio, PBC, Boston, MA, US). Data entry was performed in Microsoft Excel (Microsoft Corporation, Redmond, WA, US).

Ethical considerations

The study was approved by the Healthworld Hospital Institutional Review Board (IRB/HWHD/ACS/2025/009). As it was retrospective and based on anonymized records, informed consent was waived.

## Results

In this study, a total of 213 patients were enrolled who met the inclusion and exclusion criteria, with a median age of 58 years (IQR: 53-69 years) and 138 (64.8%) male subjects. Table 1 demonstrates the baseline clinical characteristics of the study population. The two commonest comorbidities prevalent in the study cohort were dyslipidemia (119; 55.9%) and hypertension (103; 48.4%). Non-ST elevation myocardial infarction (NSTEMI) (86; 40.4%) was the most common presentation, followed by unstable angina (UA) (73; 34.3%) and ST elevation myocardial infarction (STEMI) (54; 25.4%). Notably, 128 (60.1%) had hypotension/shock, and arrhythmia was observed in 32 (15%).

**Table 1:** Baseline clinical characteristics of the study population Data are presented as n (%).

Parameter	Number (Frequency)
Age (Median + IQR)	58 (53 – 69)
Male gender	138 (64.8%)
Risk Factors/Comorbidities	
Hypertension	103 (48.4%)
Dyslipidemia	119 (55.9%)
Current smoking	69 (32.4%)
Chronic obstructive pulmonary disease (COPD)	54 (25.4%)
Diabetes mellitus (DM)	74 (34.7%)
Past history of coronary revascularization	11 (5.2%)
Clinical Profile	
ST elevation myocardial infarction (STEMI)	54 (25.4%)
Non-ST elevation myocardial infarction (NSTEMI)	86 (40.4%)
Unstable angina (UA)	73 (34.3%)
Hypotension/shock at presentation	128 (60.1%)

The study analyzed coronary anatomy and disease patterns in 213 subjects, and this is represented in Table 2. A right-dominant coronary system was most common (146; 68.5%), followed by codominance (38; 17.8%) and left dominance (29; 13.6%). Among obstructive CAD cases, single-vessel disease (115; 54%) was most frequent, while double-vessel disease (26; 12.2%) and triple-vessel disease (29; 13.6%) were less common. Non-obstructive epicardial coronary arteries (43; 20.2%), spontaneous coronary artery dissection (SCAD) (11; 5.2%), and slow-flow phenomenon (61; 28.6%) were also observed. In 170 cases with obstructive CAD, the left anterior descending artery (LAD) was the most frequent culprit vessel (85; 50%), followed by the right coronary artery (RCA) (62; 36.5%) and with 23 (13.5%) having the left circumflex artery (LCX) as the culprit.

**Table 2:** Angiographic characteristics of the study population Data are presented as n (%).

Characteristics	Number (Frequency)
Left-dominant system	29 (13.6%)
Right-dominant system	146 (68.5%)
Codominant system	38 (17.8%)
Triple vessel disease (TVD)	29 (13.6%)
Double vessel disease (DVD)	26 (12.2%)
Single vessel disease (SVD)	115 (54%)
Nonobstructive epicardial coronary arteries (NOCA)	43 (20.2%)
Spontaneous coronary artery dissection (SCAD)	11 (5.2%)
Slow flow phenomenon	61 (28.6%)
Culprit lesion (out of 170 cases having obstructive CAD)	
Left anterior descending (LAD)	85(50%)
Left circumflex artery (LCX)	23 (13.5%)
Right coronary artery (RCA)	62(36.5%)

**Figure 1:**
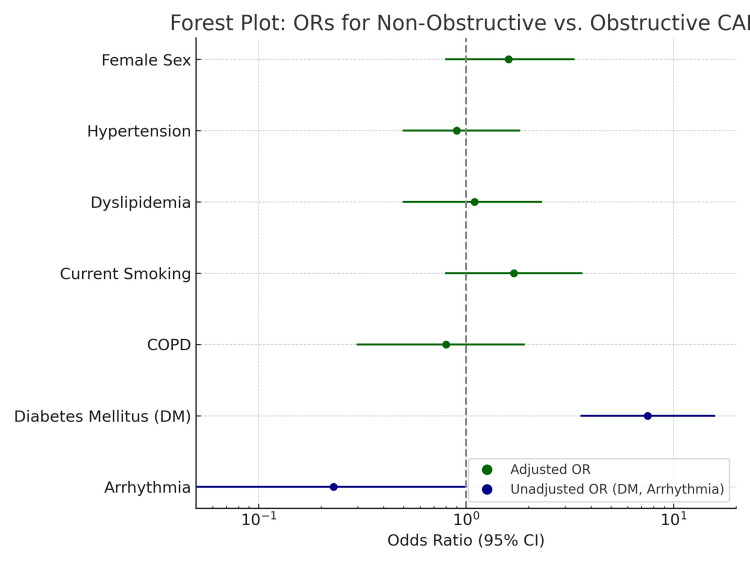
Adjusted and unadjusted odds ratios for predictors of non-obstructive vs. obstructive CAD Forest plot displaying odds ratios (ORs) and 95% confidence intervals for predictors of non-obstructive versus obstructive coronary artery disease (CAD). Green markers represent ORs adjusted for age and sex; Blue markers indicate unadjusted ORs for diabetes mellitus and arrhythmia, where adjusted models were not feasible. A logarithmic scale is used for the accurate interpretation of effect sizes. Horizontal lines denote the 95% confidence intervals. The vertical dashed line at OR = 1 represents the null value, indicating no association. Adjusted OR for diabetes could not be computed due to quasi-complete separation; unadjusted OR is shown. Adjusted analysis for arrhythmia was not feasible due to sparse event counts; unadjusted OR is presented.

In this study, 11 patients (5.2%) had SCAD as the cause of ACS. Of these, almost half (5 out of 11) were male. Patients with obstructive CAD were significantly older (median 62 vs. 44 years, p < 0.001). Diabetes was more prevalent among those with non-obstructive CAD (72.1% vs. 25.3%), yielding an unadjusted OR of 7.5 (95% CI: 3.6-15.7; p < 0.001); however, adjusted models were unstable and not included in multivariable analysis. Arrhythmia was more common in obstructive CAD (17.6% vs. 4.7%, p = 0.03). Smoking showed a trend toward higher prevalence in non-obstructive CAD (44.2% vs. 29.4%, p = 0.07), while hypertension and dyslipidemia did not differ significantly between groups. Age and diabetes status were the strongest univariate discriminators between CAD types. Table 3 and Figure 1 summarize these comparisons.

**Table 3:** Comparative analysis of non-obstructive vs. obstructive CAD Data are presented as n (%), and p < 0.05 is considered significant. † Hodges-Lehmann estimator for median difference ‡ Adjusted for age and sex. Adjusted OR for diabetes could not be calculated due to quasi-complete separation and model instability. Adjusted OR for arrhythmia was not estimated due to sparse event counts and quasi-complete separation. CAD: coronary artery disease

Characteristic	Non-obstructive CAD (N = 43)	Obstructive CAD (N = 170)	P-value	Effect Size (95% CI)	Adjusted OR‡ (95% CI)	Test Statistic
Female Sex	20 (46.5%)	55 (32.4%)	0.12	OR = 1.8 (0.9–3.6)	1.6 (0.8–3.3)	χ² = 2.43
Age (Median (IQR))	44 (41–46)	62 (55–67)	<0.001	Median = 18† (15–21)	1.22 (1.16–1.28)	N/A
Hypertension	21 (48.8%)	82 (48.2%)	0.94	OR = 1.0 (0.6–1.7)	0.9 (0.5–1.8)	χ² = 0.00
Dyslipidemia	26 (60.5%)	93 (54.7%)	0.61	OR = 1.3 (0.6–2.6)	1.1 (0.5–2.3)	χ² = 0.26
Current Smoking	19 (44.2%)	50 (29.4%)	0.07	OR = 1.9 (0.9–3.8)	1.7 (0.8–3.6)	χ² = 2.78
COPD	10 (23.3%)	44 (25.9%)	0.73	OR = 0.9 (0.4–1.9)	0.8 (0.3–1.9)	χ² = 0.12
Diabetes Mellitus (DM)	31 (72.1%)	43 (25.3%)	<0.001	OR = 7.5 (3.6–15.7)	Model unstable‡	χ² = 36.86
Arrhythmia	2 (4.7%)	30 (17.6%)	0.03	OR = 0.23 (0.05–0.99)	—	χ² = 4.51

**Figure 2:**
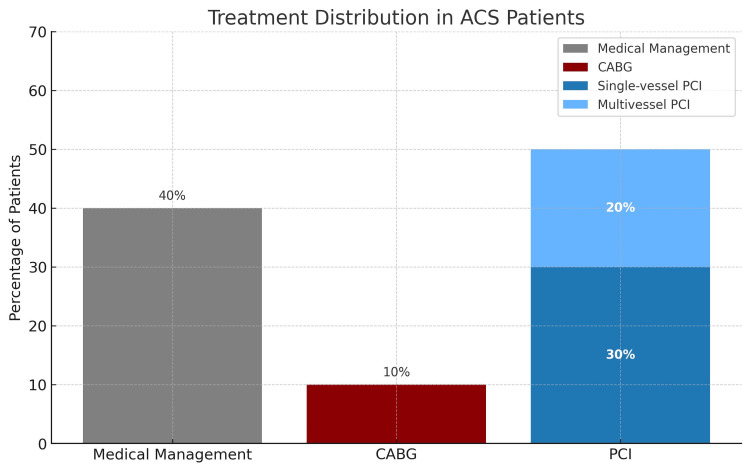
Proportional distribution of treatment strategies in patients with acute coronary syndrome Bar chart showing the distribution of management approaches among patients diagnosed with acute coronary syndrome (ACS). Medical management accounted for 40% of cases, while 10% underwent coronary artery bypass grafting (CABG). Among the 50% who received percutaneous coronary intervention (PCI), 60% underwent single-vessel PCI and 40% underwent multivessel PCI. Data reflect real-world treatment patterns in an industrial-region cohort.

The majority of patients in this study underwent percutaneous coronary intervention (PCI) (103; 48.4%), of which 78 (75.7%) patients underwent single-vessel PCI, 86 (40.4%) patients were discharged on medical management, whereas 24 patients underwent coronary artery bypass grafting (CABG). Figure 2 describes the treatment characteristics of the study population.

## Discussion

Summary of findings

This study provides a comprehensive analysis of 213 ACS patients from an industrial region in Eastern India, offering critical insights into the interplay between environmental exposures and cardiovascular disease manifestations. The cohort, with a median age of 58 years and male predominance of 138 (64.8%), exhibited a high prevalence of traditional risk factors, including dyslipidemia 119 (55.9%) and hypertension 103 (48.4%), consistent with metabolic patterns observed in rapidly industrializing regions [8]. The clinical presentation distribution showed NSTEMI (86; 40.4%), unstable angina (73; 34.3%), and STEMI (54; 25.4%). This reflects global ACS trends, though with a notably higher proportion of hypotensive presentations (128; 60.1%) as compared to Western registries [9,10]. Angiographic findings revealed several distinctive patterns: obstructive CAD patients were significantly older (median 62 vs 44 years, p<0.001) demonstrated a higher prevalence of diabetes in non-obstructive CAD (OR=7.5, p<0.001), contrary to typical obstructive CAD associations [11]. The substantial proportion of non-obstructive CAD 43 (20.2%) and slow-flow phenomenon 61 (28.6%) suggests significant microvascular involvement, potentially related to chronic particulate matter exposure [12]. Most remarkably, the SCAD prevalence 11 (5.2%) included equal male-female distribution, dramatically differing from the 80-90% female predominance reported in contemporary SCAD registries [13,14].

Comparison with contemporary studies

Our demographic findings align closely with Indian registry data (CREATE: mean age 56; Kerala ACS: mean age 60) [5,6], but contrast sharply with Western cohorts (GRACE: median age 66; SWEDEHEART: 30% female) [15,16]. This discrepancy may reflect regional variations in CAD onset patterns, genetic susceptibility, and environmental exposures [17].

The high prevalence of non-obstructive CAD and slow-flow phenomenon exceeds typical Western reports (10-15%) but matches emerging data from heavily polluted Asian cities [18]. These findings support the environmental vasomotor dysfunction hypothesis, where chronic particulate matter exposure induces endothelial dysfunction and microvascular impairment [19,20]. Recent mechanistic studies have demonstrated PM2.5-induced coronary vasoconstriction and impaired flow-mediated dilation, potentially explaining our observations [21,22].

The observed association between diabetes and non-obstructive CAD in our cohort, while statistically significant in unadjusted analysis, became unstable upon multivariable adjustment due to quasi-complete separation and collinearity. We therefore report only the unadjusted OR and interpret this finding with caution. Similar patterns have been noted in the INTERHEART South Asian cohort [23], suggesting that unique gene-environment or metabolic factors may influence plaque characteristics in this population. However, the possibility of selection bias and model distortion cannot be excluded [24]. The equal sex distribution in SCAD cases challenges current understanding and parallels recent reports from industrial regions in China, suggesting unidentified environmental or occupational triggers in male patients [25].

Implications for future research

These findings highlight several critical research directions. First, prospective studies should incorporate detailed environmental monitoring (PM2.5, heavy metals) with serial vascular function assessments to establish causal relationships [26]. The European ESCAPE study framework could be adapted for industrial regions [27].

The unexpected finding of higher diabetes prevalence in non-obstructive CAD warrants further exploration. While this may partly reflect selection bias or model limitations, prior experimental studies suggest that exposure to particulate matter can alter insulin signaling and influence plaque characteristics [28]. Although speculative, such pollution-modified metabolic pathways could contribute to atypical CAD presentations in high-risk environments and merit exploration in future studies. Third, the high male SCAD prevalence necessitates dedicated studies evaluating occupational exposures (vibration, heavy lifting) and pollution-induced vascular wall changes.

Methodologically, future work should include: coronary physiology assessment (coronary flow reserve (CFR), index of microcirculatory resistance (IMR)) in slow-flow cases [29]; plaque characterization (optical coherence tomography (OCT), intravascular ultrasound (IVUS)) in diabetic vs non-diabetic obstructive CAD; long-term follow-up comparing industrial vs non-industrial ACS populations

Study limitations 

This single-center study, while informative, has several limitations. Generalizability may be limited, although the cohort reflects the region’s industrial demographic [30]. Selection bias is possible, as only patients who underwent coronary angiography were included, excluding those with atypical symptoms, conservative management, or denied/refused invasive evaluation. All cardiology-admitted ACS patients underwent angiography, and none died prior to the procedure. However, we lacked triage-level exclusion data, and patients who died before admission or declined angiography were not captured, potentially underrepresenting severe ACS cases and contributing to workup bias.

The use of the conventional STEMI-NSTEMI classification may inadequately represent the pathophysiological diversity of ACS. As noted by the reviewer, adopting the OMI-NOMI (occlusion MI vs. non-occlusion MI) framework in future studies could offer a more precise stratification.

Additional limitations include the absence of invasive physiological assessment (e.g., fractional flow reserve (FFR), IMR), short-term follow-up, and lack of data on environmental exposures, inflammatory markers, or genetic susceptibility, factors that could clarify the mechanistic link between industrial pollution and CAD phenotype.

## Conclusions

Our study reveals distinct ACS patterns in an industrial region, featuring high microvascular dysfunction rates and atypical SCAD presentations. These findings underscore the need for environmental cardiology frameworks and region-specific management protocols. Future research must integrate pollution monitoring with advanced vascular phenotyping to unravel these complex interactions.
