# Correction: Clinical and Angiographic Profile of Acute Coronary Syndrome: Insights From a Tertiary Care Center in an Industrial Area

**DOI:** 10.7759/cureus.c230

**Published:** 2025-07-11

**Authors:** Anindya Banerjee, Ishita Majumdar, Madhurima Ganguly, Mohammed I Hussain, Debasish Das

**Affiliations:** 1 Cardiology, Healthworld Hospital Asansol, Asansol, IND; 2 Neonatology, Institute of Post Graduate Medical Education and Research (IPGMER) and Seth Sukhlal Karnani Memorial Hospital (SSKM Hospital), Kolkata, IND; 3 Cardiology, All India Institute of Medical Sciences, Bhubaneswar, Bhubaneswar, IND

This article has been corrected by the Editor-in-Chiefs to replace language that could be considered misleading. Specifically, the term “paradoxical inverse association” was a mischaracterization. The unadjusted OR of 7.5 (95% CI: 3.6–15.7; p<0.001) indicates a positive association between diabetes mellitus and non-obstructive CAD (i.e., diabetes was more prevalent in the non-obstructive group). Referring to this as an "inverse association" was incorrect, and using the term "paradox" was misleading. Three instances of this language found in the Abstract and Discussion sections have been revised.

